# Microinjection of Ghrelin into the Ventral Tegmental Area Potentiates Cocaine-Induced Conditioned Place Preference

**DOI:** 10.4236/jbbs.2013.38060

**Published:** 2013-12

**Authors:** Lindsey M. Schuette, Christopher C. Gray, Paul J. Currie

**Affiliations:** Department of Psychology, Reed College, Portland, USA

**Keywords:** Cocaine, Conditioned Place Preference, Ghrelin, Psychostimulant, Midbrain, Ventral Tegmental Area

## Abstract

Prior work has shown that systemic cocaine pretreatment augments cocaine conditioned place preference (CPP) in rats. In contrast, ghrelin receptor antagonism attenuates cocaine and amphetamine-induced CPP. In order to further investigate ghrelin’s role in dopamine-mediated reward, the present report examined whether pretreament with ghrelin, administered directly into the ventral tegmental area (VTA) of the midbrain, would potentiate the rewarding properties of cocaine as measured by CPP. Adult male Sprague-Dawley rats were given access to either side of the CPP chamber in order to determine initial side preferences. The rats were then restricted to either their non-preferred or preferred side over the course of conditioning which lasted for a total of 16 consecutive days. This was followed by a final test day to then reassess preference. On days where rats were confined to their non-preferred side, ghrelin (30 – 300 pmol) and cocaine (0.625 – 10 mg/kg IP) were administered immediately prior to the conditioning trial. On alternate days rats were treated with vehicle and placed into what was initially determined to be their preferred side. CPP was calculated as the difference in percentage of total time spent in the treatment-paired compartment during the post-conditioning session and the pre-conditioning session. Our results indicated that both cocaine and ghrelin elicited CPP and that ghrelin pre-treatment potentiated the effect of cocaine on place preference. Overall, these findings provide additional support for the argument that ghrelin signaling within the VTA enhances the rewarding effects of psychostimulant compounds.

## 1. Introduction

Extensive evidence implicates ghrelin, a 28-amino acid acylated peptide, in the hypothalamic regulation of food intake and energy metabolism [1–4]. The stomach-derived peptide is also involved in the neural control of stress and anxiety-like behavior [5–8], memory [9–10] as well as reward mechanisms of the mesolimbic dopamine system [11–14]. In fact ghrelin targets neurons of the ventral tegmental area (VTA) and nucleus accumbens to increase appetitive motivation and other consummatory behaviors such as ethanol self-administration [12,15]. In this regard, systemic ghrelin, as a result of passive transport across the blood-brain barrier, may bind to receptors distributed throughout the brain, and in particular, those expressed in the VTA [16,17]. Prior work has also shown that peripheral ghrelin sensitizes cocaine-stimulated hyperlocomotion [18] and alters cocaine-induced conditioned place preference (CPP) [19]. Moreover, antagonism of the ghrelin 1a receptor (GHS-R1A) following intraperitoneal administration of JMV2959 attenuates cocaine and amphetamine-induced locomotor activity in addition to dopamine-stimulated release in the nucleus accumbens [20]. In this same study, the ability of either cocaine or amphetamine to elicit CPP was attenuated by JMV2959.

In order to further investigate the role of ghrelin signaling in mediating the rewarding properties of psychostimulant drugs, in the present study we examined the impact of VTA ghrelin pretreatment on cocaine-induced CPP. Our findings, described previously in preliminary form [21], demonstrate that direct ghrelin injections into the VTA potentiate place preference elicited by threshold doses of cocaine and induce CPP when paired with sub-threshold dosing.

## 2. Materials and Methods

### 2.1. Animals

Adult male Sprague-Dawley rats (N = 128, Harlan) weighing 275 to 325 g at the time of surgery were used in all experiments. Animals were pair-housed and allowed to habituate to the colony environment for three weeks prior to surgery and then individually housed post-surgery in hanging polypropylene cages with free access to food (LabDiet) and water. The room was maintained on a 12 h light/dark cycle (lights off at 1400 h) at a constant temperature of 22 ± 2°C. Testing was conducted during the active/dark cycle. All experiments were carried out in accordance with the Institutional Animal Care and Use Committee guidelines of Reed College.

### 2.2. Stereotaxic Surgery

Animals were anesthetized with pentobarbital sodium (50 mg/kg IP). Supplemental ketamine (15 mg/kg, SC) was administered as needed throughout surgery. All rats were placed into a Kopf stereotaxic frame with the incisor bar set to 3.5 mm below the interaural line. Stainless steel guide cannulae (22 gauge; Plastics One, Roanoke, VA) were unilaterally implanted 4 mm dorsal to the VTA with placements counterbalanced across hemispheres. Stereotaxic coordinates relative to bregma were posterior 5.3 mm; lateral ±1 mm; ventral 4.1 mm [22]. The cannulae were secured with three stainless steel screws and acrylic cement and fitted with stylets (30 gauge; Plastics One). Behavioral testing began after a 14-day recovery period.

### 2.3. Apparatus

Place conditioning and testing were conducted in rectangular PVC boxes (68 × 21 × 21 cm; MED Associates) divided into three chambers with transparent plexiglass ceilings. Enclosure doors set into the walls separated the three chambers and could be locked in the open or closed position for conditioning sessions. The smaller centre chamber (12 × 21 × 21 cm) consisted of gray walls and a solid plastic gray floor. The two larger chambers (28 × 21 × 21 cm) on either side of the centre chamber were comprised of either solid black or white walls with parallel or crosshatched metal bars for flooring. Pre-conditioning, conditioning, and post-conditioning sessions were digitally recorded under low light conditions as previously described [19] and no other illumination was provided in the test room.

### 2.4. Experimental Protocol

Acylated rat ghrelin (Tocris) was dissolved in sterile isotonic saline and injected unilaterally into the VTA with a 32-gauge microinjector (Plastics One). The injector extended 4 mm beyond the end of the implanted cannula with the injector tip ending 8.1 mm ventral from the skull surface. A total volume of 0.2 μl was delivered over a two-minute period with the injector left in place for an additional one minute to permit the diffusion of the peptide into the VTA. Cocaine hydrochloride (Sigma) was dissolved in sterile isotonic saline and injected intraperitoneally (IP). Conditioning and testing were conducted over an 18-day period and consisted of a preconditioning day, 8 CS+ days alternating with 8 CS− days, and a post-conditioning day.

#### Pre-conditioning

A preconditioning test trial was conducted on the first day of the experiment to test for initial chamber preference. Rats were weighed and mock injected and then placed into the centre chamber of the CPP apparatus with the doors to both adjoining chambers locked in the open position to allow free movement. Behavior was remotely recorded for 20 minutes and later analyzed for time spent in each chamber. The less preferred chamber for each individual animal was assigned as the CS+ chamber for future conditioning sessions. Rats were removed and replaced in their home cages.

#### Conditioning Trials

On 8 alternating days (days 2, 4, 6, 8, 10, 12, 14, and 16) animals were administered ghrelin or vehicle into the VTA, paired with vehicle or cocaine (0.625, 2.5 or 10 mg/kg IP). In one experiment ghrelin was administered at a dose of 300 pmol and in a separate experiment the ghrelin dose was reduced to 30 pmol. Immediately after injections, rats were placed in the CS+ chamber (the compartment corresponding to their less-preferred chamber as determined in the preconditioning test session). The doors to the adjoining compartments were locked in the closed position so that the rat only had access to the CS+ chamber. Rats were removed after 20 minutes and returned to their home cages. The procedure on the 8 intervening days (days 3, 5, 7, 9, 11, 13, 15, and 17) was identical to that of ghrelin-cocaine treatment days, except that all animals received vehicle injections, and were then placed into the CS3− chamber.

#### Post-conditioning

In each experiment post-conditioning testing mirrored the pre-conditioning protocol. Rats were given free access to both chambers of the apparatus and their behavior was digitally recorded over 20 min.

### 2.5. Histological and Statistical Analyses

Cannula placements were confirmed via histological examination. Tissue sections were examined by light microscopy and viewed relative to the stereotaxic atlas of Paxinos and Watson [22]. Data were analyzed using two-way between groups analyses of variance (ANOVA) followed by a Bonferroni test for group comparisons. The criterion for statistical significance was p < 0.05.

## 3. Results

**Figure 1:**
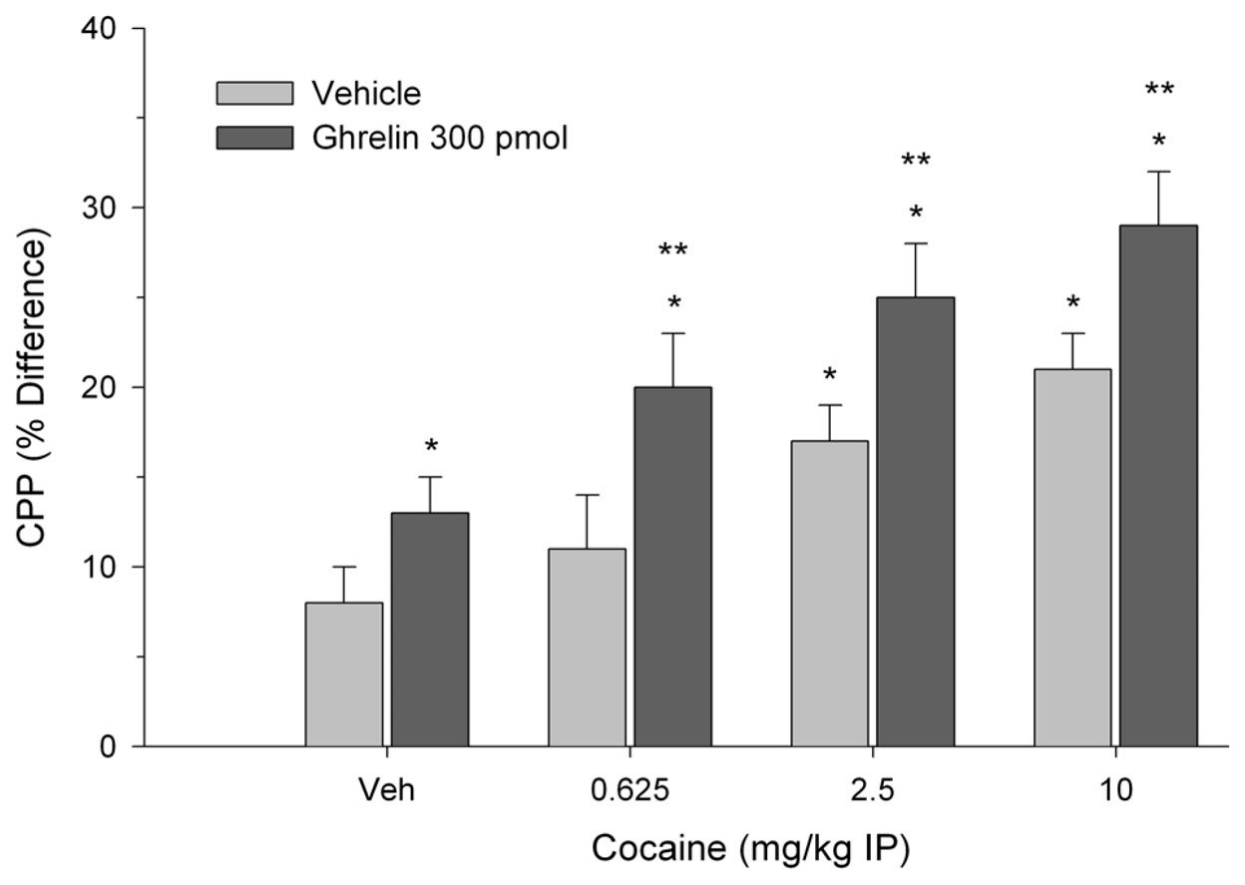
Effect of 300 pmol of ghrelin injected directly into the VTA and paired with systemic cocaine on place preference. CPP is expressed as the difference in percentage of total time spent in the treatment-paired compartment during the post-conditioning session and pre-conditioning session. Values represent mean ± S.E.M. ^*^p < 0.05 compared to vehicle-vehicle (Veh/Veh). ^**^p < 0.05 compared to cocaine paired with Veh.

**Figure 2:**
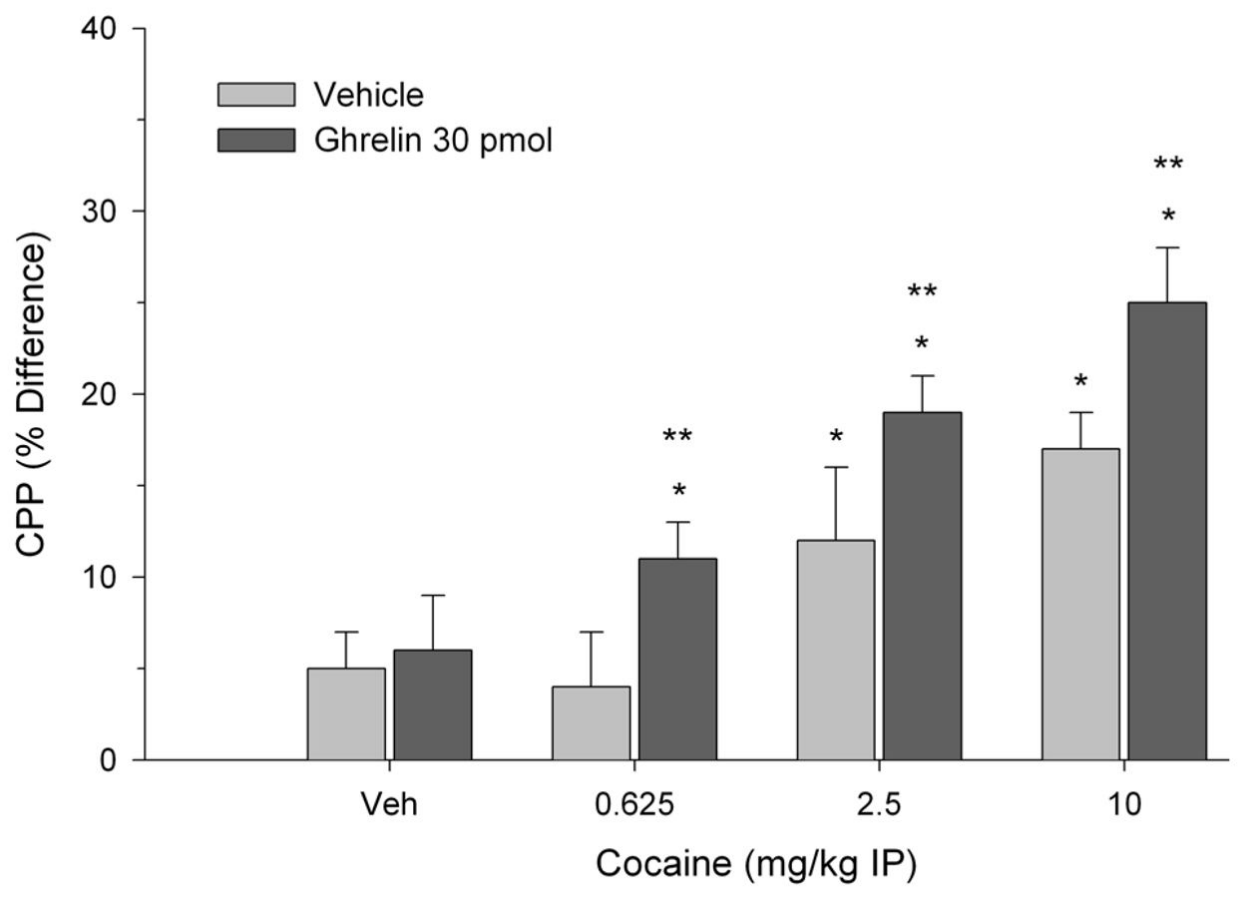
Effects of ghrelin administered at a dose of 30 pmol and paired with cocaine treatment on CPP. Values are represented as mean (± S.E.M.) CPP scores and reflect the difference in percentage of total time spent in the treatment-paired compartment during the post-conditioning session and pre-conditioning session. ^*^p < 0.05 compared to Veh/Veh control. ^**^p < 0.05 compared to cocaine paired with Veh.

CPP was calculated as the difference in percentage of total time spent in the treatment-paired (least preferred) compartment during the post-conditioning session and pre-conditioning session as described previously [20]. In each experiment between groups analyses of variance indicated a significant two-way interaction. Specifically, as shown in Figure 1, both cocaine and ghrelin (300 pmol) treatment elicited CPP when paired with vehicle. The effective doses of cocaine were 2.5 and 10 mg/kg but the lowest dose of 0.625 mg/kg was ineffective. Moreover, 300 pmol of ghrelin potentiated CPP in rats treated with threshold doses of cocaine and induced CPP when paired with the subthreshold cocaine dose (0.625 mg/kg) (two-way interaction, F(3.56) = 9.12, p < 0.001). In a second experiment, as illustrated in Figure 2, 30 pmol of ghrelin injected into the VTA failed to induce CPP. However, this same dose of ghrelin reliably potentiated CPP elicited by 2.5 and 10 mg/kg of cocaine (two-way interaction, F(3.56) = 3.92, p < 0.02). As in the previous experiment, when ghrelin was paired with 0.625 mg/kg of cocaine, place preference was induced.

## 4. Discussion

The focus of the present study was to investigate the impact of ghrelin and cocaine co-administration on the conditioning of place preference. Both experiments demonstrated that ghrelin microinjection directly into the mesolimbic VTA augmented CPP elicited by cocaine. Additionally a subthreshold dose of ghrelin was effective in either potentiating CPP evoked by threshold doses of cocaine or in inducing place preference when co-injected with a subthreshold cocaine dose. Given that ghrelin receptors are localized to the VTA and that prior work has shown that systemic ghrelin increases dopamine turnover in the nucleus accumbens [17,23], this suggests that expression of ghrelin receptors on VTA dopamine neuronal projections provides a mechanism through which ghrelin signaling modulates the reinforcing properties of psychostimulant drugs like cocaine.

In related work, exogenous ghrelin administration has been reported to increase appetitve motivation including operant responding for food [24]. VTA injection of the dopaminergic neurotoxin 6-OHDA attenuates this action indicating that ghrelin’s effect on appetitve motivation requires intact mesolimbic dopamine transmission [24]. Consistent with this argument is additional evidence demonstrating that ethanol-induced locomotor stimulation and accumbal dopamine release are both suppressed in ghrelin knockout mice [25] suggesting that endogenous ghrelin is required in order for ethanol to activate the mesolimbic dopamine system. Moreover, ghrelin and ghrelin 1a receptor activity are required for the induction of locomotor sensitization to cocaine [26,27]. If rats are injected with the 1a antagonist JMV2929 prior to cocaine treatment, these animals exhibit significantly impaired cocaine-induced hyperlocomotion. Ghrelin receptor knock out rats also exhibit diminished development of cocaine-induced locomotor sensitization compared to wild-type controls [27].

Overall the above work is consistent with emerging evidence implicating mesolimbic ghrelin signaling in drug reward, specifically via an action on ghrelin 1a receptors expressed by ventral tegmental dopamine neurons. Ghrelin increases the intake of rewarding foods [24,28,29] and additional work suggests that dopamine VTA-accumbal projections mediate the peptide’s effects on food reward and not simply on the intake of standard rodent chow [15]. Specifically, pretreatment with dopaminergic receptor D1/D2 antagonists injected into the nucleus accumbens blocks the stimulatory effect of ghrelin on sucrose intake without altering chow intake. Such findings underscore the importance of interacting ghrelin and dopamine mechanisms within mesolimbic neurons as critical in the mediation of rewarding and motivated behavior.

## 5. Conclusion

Direct VTA injections of ghrelin elicited CPP and potentiated or induced place preference elicited by administration of cocaine. The effect occurred at both threshold and subthreshold doses. Overall our findings are consistent with increasing evidence indicating that ghrelin plays a critical role in central mechanisms related to reward signaling and specifically in augmenting the action of psychostimulant compounds within the mesolimbic dopamine system.
